# Surface related laser induced white emission of Cr:YAG ceramic

**DOI:** 10.1038/s41598-021-93638-2

**Published:** 2021-07-07

**Authors:** M. Chaika, R. Tomala, W. Strek

**Affiliations:** Institute of Low Temperature and Structure Research, Polish Academy of Science, 50-422, Wrocław, Poland

**Keywords:** Applied optics, Lasers, LEDs and light sources, Optical materials and structures

## Abstract

In this work we report the white light emission in transparent Cr:YAG ceramic pellet upon irradiation with focused beam of CW infrared laser diode. It was found that this phenomenon is specifically related to interaction of laser beam with a surface of the pellet. The white light was emitted outside an irradiated spot at the surface of the pellet and did not penetrate inside the pellet. Moreover, the red emission related to two-photon absorption along the laser beam penetrating the Cr^3+^:YAG pellet was observed. Interaction of the laser beam with the surface of the pellet leads to an efficient white light emission from an outer side of the pellet. The resulting white light emission did not entry back the pellet. Multiphoton ionization leading to intervalence charge transfer followed by light emission was proposed as the mechanism of experimentally observed white light emission.

## Introduction

Laser induced white emission (LIWE) in rare earth doped oxides excited by focused laser beam in vacuum was reported by Wang and Tanner^1^. Since then, LIWE was observed in different materials placed in vacuum ambient^2–8^. An increase in the ambient pressure was accompanied by a decrease of LIWE intensity, which remained stable up to critical point^9,10^. The excitation power dependence of LIWE intensity demonstrated a threshold behavior for multiphoton avalanche processes^1^. Numerous models have been proposed for white light generation in lanthanide-based systems including the intervalence charge transfer (IVCT)^9,11^, multiphoton absorption^12^, thermal avalanche^13^ and others^2,14^.

Recently we have reported LIWE in transparent Cr:YAG ceramics^15,16^. In a course of studies we have found that an occurrence of white emission was closely related to the interaction of focused laser beam with the surface of ceramics. In the present study we study the LIWE process in Cr:YAG transparent ceramics under CW IR laser excitation. It was shown that LIWE is the surface related phenomenon and the light is emitted only out of the interaction spot of laser beam and does not penetrate the ceramics.

## Experimental

The Cr^4+^:YAG ceramics was made in Institute for Single Crystals, Kharkiv, Ukraine. The Cr:YAG samples were sintered in vacuum furnace by solid state reaction. High purity High purity reagents: Al_2_O_3_ (purity > 99.99%, Baikowski, d = 0.15–0.3 μm), Y_2_O_3_ (purity > 99.999%, Alfa Aesar, d =  < 10 μm), Cr_2_O_3_ (purity > 99.97%, Alfa Aesar, d =  < 100 nm), CaO (purity > 99.999%, Sigma Aldrich, d =  < 0.1 μm) were used as starting materials. Powders were taken in stoichiometric ratio, concentrations of Ca and Cr were taken in order to replace Y and Al, respectively. Cr_2_O_3_ and CaO powders were weighted precisely to obtain chromium and calcium content of 0.1 and 0.5 at.%, respectively^9^. Homogenization was performed by ball milling for 15 h using high purity Al_2_O_3_ balls. The milled slurry was dried for 1 day in air and sieved through a 200-mesh screen. The compacts were prepared by applying isostatic pressing at P = 250 MPa. Sintering was performed at 1750 °C for 50 h using solid state reaction (SSR) in vacuum furnace.

**Figure 1:**
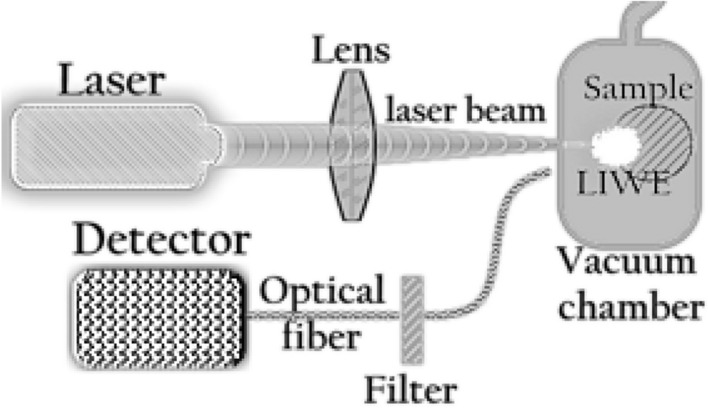
Scheme of LIWE measurement setup.

Scheme of the LIWE measurement setup is shown in the Fig. 1. The samples were placed in a vacuum cell connected to EXT75DX turbo molecular vacuum pump with TIC controller (Edwards) to achieve the pressure of 10^–4^ mbar. To collect the emission spectra, the AVS-USB2000 Avantes Spectrometer was used. Emission spectra were measured using an infrared continuous wave Nd:YAG laser 3,4 W as an excitation source. The spectra were not corrected to the detector sensitivity. In order to protect the CCD camera, FGS900S filter was used for measurement of the power dependence of LIWE intensity. LIWE was measured under P -10^–4^ Pa. The effect of pressure on the LIWE intensity of Cr:YAG ceramics was studied in our previous work^15^.

## Results and discussion

**Figure 2:**
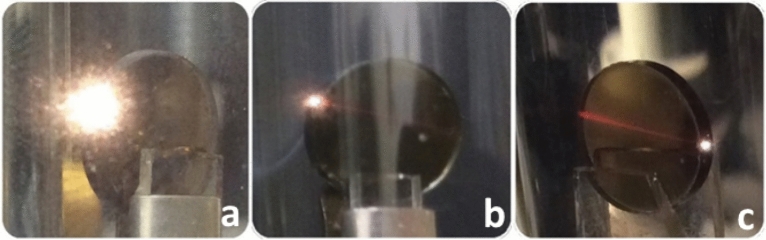
The photos of LIWE observed for Cr:YAG ceramic pellet under different: high (**a**), intermediate (**b**) and low (**c**) excitation power densities using CW 1064 nm laser focused to a spot with the diameter of 175 µm in vacuum (**c**).

The measurements of laser induced emission spectra of Cr:YAG ceramics were carried out by exciting the sample in vacuum in different places with a focused laser beam. Round Cr:YAG ceramics pellets with thickness of 1 mm and diameter of 8 mm were taken. The experiments demonstrated that the characteristics of the emission resulting from irradiation of the ceramic pellet depended on the excitation laser power density on the illuminated pellet surface. The photos demonstrating interaction of laser beam with the edge of the pellet for different excitation power densities are shown in Fig. 2. The experiments were also performed for the rectangular sample plate ceramic (see Fig. S1 in Supplementary).

In experiment we have used a focused laser beam with a diameter of excitation spot of 175 µm. For 3.4 W laser power the excitation power density is close to ~ 10^8^ W/m^2^^16^. In a course of experiments it was found that Cr:YAG ceramics is able to generate the bright LIWE for excitation power density threshold of 0.7 W for focused CW laser beam (1064 nm)^15,16^. Intense LIWE can be generated at relatively low temperature of the sample (below 50 °C)^4^. Therefore, the sample temperature can not be responsible for white colour black body emission. LIWE intensity depends sufficiently on the excitation density. A tuning of the laser beam by several tens of micrometers can cause a decrease in the emission intensity by an order of magnitude or its complete disappearance. However, the intense LIWE can be successfully observed over an entire surface of the sample.

**Figure 3:**
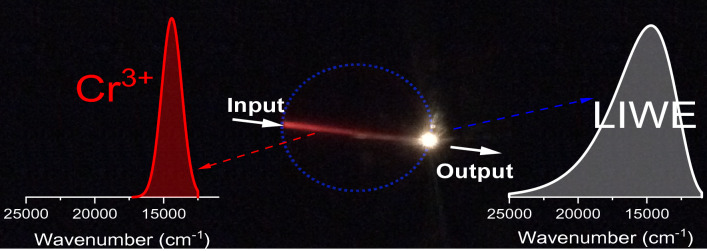
The photo of Cr:YAG ceramics under 1064 nm laser excitation in vacuum and emission spectra observed under focused 1064 nm laser beam. The blue circle indicates the shape of the sample.

The laser path highlighted in red in the Fig. 2 is caused by Cr^3+^ emission. When the excitation light was focused on a surface of the sample and its power exceeded certain threshold, the samples generated LIWE. The LIWE was observed on both unpolished (see Fig. 2) and polished (see Fig. S2 in Supplementary) surfaces of the sample. The average intensity of LIWE on the unpolished side was higher than on the polished one. The SEM images of polished and unpolished surfaces have been added to the paper (see Fig. S6). It suggests that the intensity of LIWE depends on the surface roughness. Moreover, when the incident laser beam spot was larger than the spot of incident focused laser beam, the white light emission was not observed. The laser beam entered the pellet leading to red emission with the spectrum assigned to the ^4^T_2*g*_* → *^4^A_2*g*_ transitions of Cr^3+^ ions (see Fig. 3). Beforehand, it was shown that when the Cr^3+^:YAG single crystal was irradiated by a focused infrared laser, strong red emission was observed along direction of irradiation in the volume of the sample^12^.

The broadband emission spectra for different excitation powers were reported by us earlier^12^, LIWE spectra under 3.4 W of 1064 nm excitation see on Fig. S5. The excitation power density of the focused laser beam at the excitation spot with diameter D = 0.175 mm was estimated to be 10^8^ W/m^2^. This value is consistent with the thickness of the red line (0.16 ± 0.03 mm) near the beam entry where LIWE is generated. The shift of the focus deeper into the sample leads to decrease in the excitation density and the disappearance of the white emission at the entry point and, at the same time, increase in the power density at the exit point and the appearance of the white emission after exceeding the threshold. In addition, self-focusing leads to additional focusing of the laser beam in the bulk (see Fig. 3 and relevant text in Supplementary).The thickness of the red line at the entry and exit points is 0.34 ± 0.03 mm and 0.11 ± 0.03 mm, respectively (Fig. S3).

**Figure 4:**
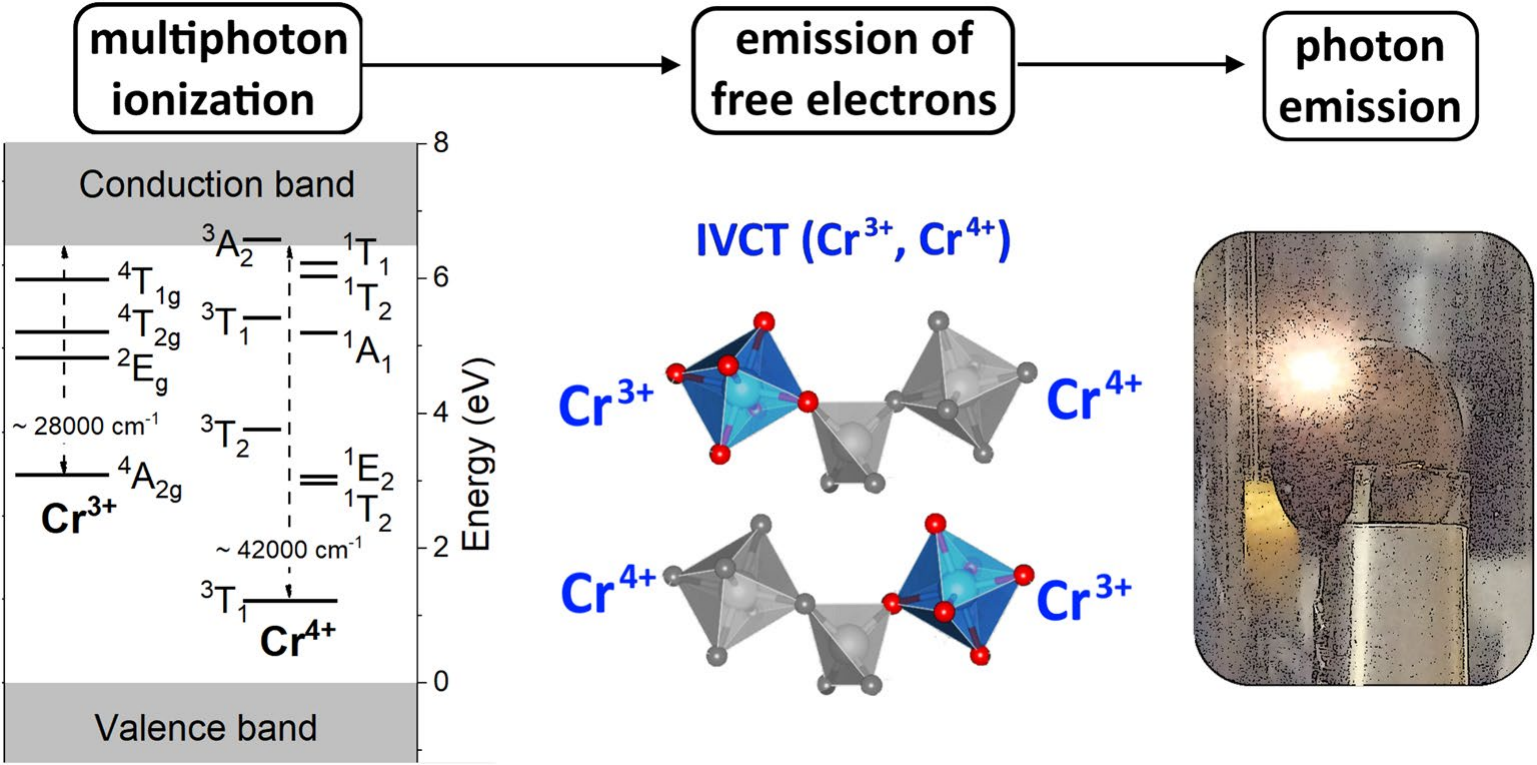
Schematic illustration of the mechanism responsible for LIWE from the transparent Cr^4+^:YAG ceramics.

The interaction of the laser beam with the surface of the sample leads to ionization with simultaneous emission of photons and free photoelectrons^17^. Emission of free electrons is preceded by the multiphoton (N-photon) absorption ionization process. The electron emission rate *J* related to multiphoton absorption necessary for ionization is described by a simple formula *J* $\propto$ *I*^*N*^, where *I* is the intensity of the incident laser beam^18,19^. An ionization process is more complex since apart from multiphoton absorption, an avalanche ionization plays a significant role^20^. Broadband laser induced white emission may be described by intervalence charge transfer (IVCT) transitions following the ionization of Cr^3+^ ion^15^ Cr^3+^  + *N ћω* → Cr^4+^  + e^-^ and then IVCT transitions generating the broadband laser induced white emission **ν**(LIWE) (Cr^4+^, Cr^3+^) → (Cr^3+^, Cr^4+^) + **ν**(LIWE) (see Fig. 4)^10^. A detailed explanation of the role of LIWE phenomenon in the white light emission was reported earlier^15,16^. The white light was emitted only in an irradiation spot at the side surface of pellet and was emitted off the side surface of pellet, it does not penetrate the pellet. No white light was observed inside the pellet (see Fig. S4 and the video file in the Supplement).

To conclude the emission of white light is surface related phenomenon. The following features of emitted light due to interaction of incident laser beam with the ceramic pellet can be mentioned:

1. Laser beam passing through a pellet leads to the white light emission only from the outer side of pellet.
2. LIWE occurred in opposite direction to laser beam.
3. The self-focusing occurs before the geometrical focus. Laser beam propagating through transparent medium with an effective refractive index determined by nonlinear index of refraction n_2_ (Kerr effect) n = n_0_ + n_2_ I, where I is the intensity of laser beam. The index n_2_ is positive.
4. Intensity of LIWE strongly depends on the surface roughness being higher for higher roughness.
5. Self-focused laser beam inside the ceramic approaches its edge and becomes a point source of white light emission outside. Such behavior is due to the fact that ionization responsible for white light is preceded by emission of electrons.

The surface plays an important role in inducing LIWE, however, many questions still need more elaborated studies. The IVCT mechanism does not take into account the role of the surface in the LIWE phenomenon. To discuss the role of surface we propose the following model: The laser beam interacts with the surface of the pellet leading to multiphoton ionization assisted by emission of photons and hot electrons. It is known that a number of emitted electrons is higher than a number of emitted phonons. Since electrons cannot penetrate inside the pellet, the ionization process may occur only outside the sample. The processes occurring in the system may be schematically depicted as a sequence of the following processes: multiphoton ionization of Cr^3+^ → emission of free electrons and formation of Cr^4+^  → IVCT (Cr^3+^, Cr^4+^) → **V**(LIWE). Moreover, the multiphoton ionization process can lead to generation of the plasma at the LIWE spot via ejection of free electrons preceding photon emission. In conclusion, the main statement of the present work, that LIWE is caused by the ionization process, can occur only outside the bulk, while various possible mechanisms of multiphoton absorption can be proposed for different materials^2^.

## Summary

In the present work we have investigated the laser induced white emission upon irradiation of Cr:YAG transparent ceramic pellet. It was found that the white light was emitted only on the outer side of the pellet surface from a spot irradiated by focused laser beam. The emitted white light did not penetrate inside the ceramic pellet and being emitted from the outer side of pellet surface. For slightly larger excitation spots no white light emission was observed, but only the red light emission assigned to the ^4^T_2*g*_* → *^4^A_2*g*_ fluorescence of Cr^3+^ ions due to two photon absorption of 1064 nm laser light. The transmitted laser beam demonstrated self-focusing and an intense white light emission was observed in the exit point.

## Supplementary Information

Supplementary Information.

Supplementary Video 1.
