# Supplementary material for: Surface related laser induced white emission of Cr:YAG ceramic
Source: Sci Rep. 2021 Jul 7;11:14063. doi: 10.1038/s41598-021-93638-2 (PMC8263758; doi:10.1038/s41598-021-93638-2)
Supplement: Supplementary file 1 — Supplementary Information. [file 41598_2021_93638_MOESM1_ESM.docx]

***Supplementary material***

**Surface related laser induced white emission of Cr:YAG ceramic**

M. Chaika*, R. Tomala, W. Strek

*Institute of Low Temperature and Structure Research,*

*Polish Academy of Science, 50-422 Wroclaw, Poland.*

*e-mail. m.chaika@intibs.pl*


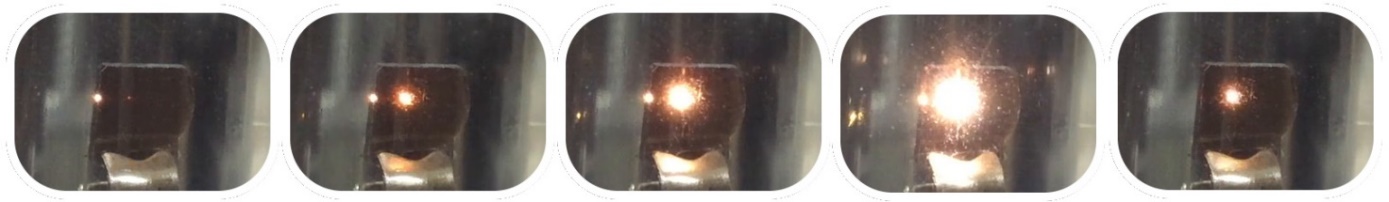


Fig. S1: The photos of LIWE observed for the rectangular sample plate Cr:YAG ceramic.

The fact that the white light is emitted only outside the ceramic pellet and does not entry back the pellet requires more detailed explanation. A possible explanation for this could be related to the plasma which probably appears during LIWE. The emitted light can be partially reflected by this plasma. However, this is based on the assumption that plasma is generated during LIWE, which is not conclusive evidence. This point is difficult to explain and, therefore, needs theoretical consideration.


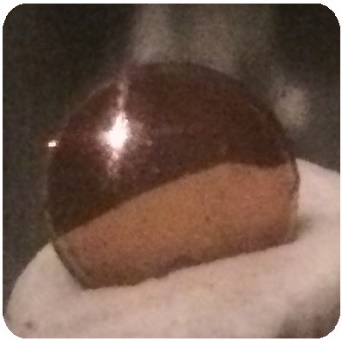


Fig. S2: The photos of Cr:YAG ceramic pellet excited by 1064 nm laser beam with smaller excitation density of side wall of pellet in vacuum.


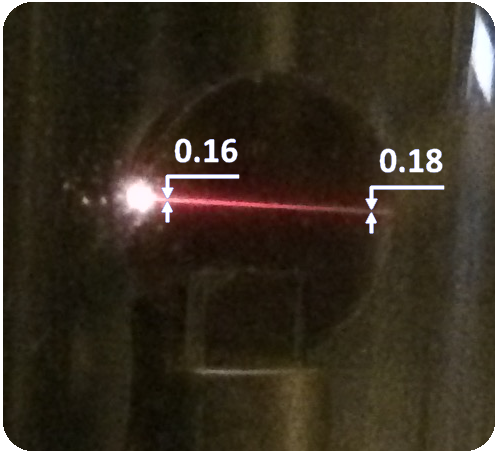

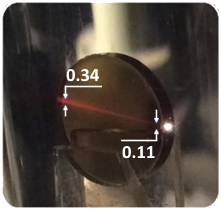


Fig. S3: The photos of LIWE observed for Cr:YAG ceramic pellet at the entry (left) and exit (right) points.

Self- focusing in transparent media is associated with nonlinear index of refraction in a Kerr medium. We suppose that the self- focusing takes a place in the volume of the sample which is helps to achieve an appropriate density for LIWE of the laser beam at the output from sample. This assumption based on the measured diameter of the laser beam. The measured diameter of the laser beam at the input point was 0.16 ± 0.03 mm which is agree with the reported earlier value for diameter of the laser beam in the focus point for the same equipment [6]. However, the estimated diameter of the laser beam at the output from the sample volume was 0.11 ± 0.03 mm which is lower than those which can be achieved by our focusing lens.


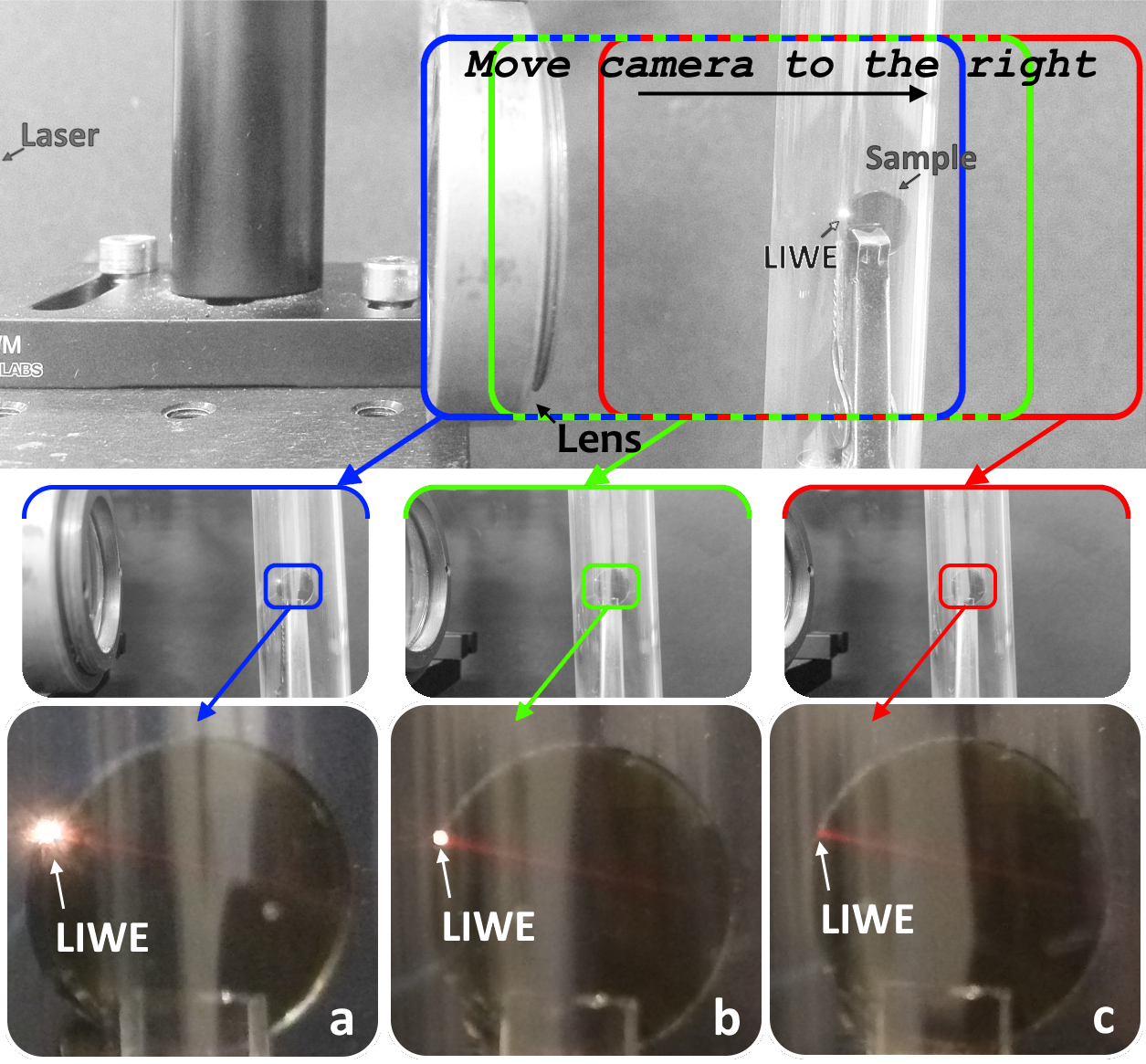


Fig. S4: The photos of LIWE of Cr:YAG ceramic under 1064 nm laser excitation in vacuum, taken from different positions, with the edge surface is a) visible, b) partially visible and c) hidden.

Fig. S4 shows the photos of Cr:YAG ceramics under 1064 nm laser excitation in vacuum taken from different positions. The photos were taken by moving the camera parallel to the side surface, so that both the edge and side surfaces were visible first (Figs. S1a and b) and then the edge surface was hidden. See Video 1 for details. When the edge surface was visible, the intense white emission can be observed on the photo (see Fig. S4a). At the same moment, the white emission isn’t visible when the edge surface was hidden (see Fig. S4c). This indicates that the LIWE generation takes only on the sample surface and do not penetrate into the sample volume.

Fig. S5:VIS (a) and NIR (b) part of LIWE spectrum of the Cr:YAG ceramics measured under focused 1064 nm laser excitation in the laser entrance point.


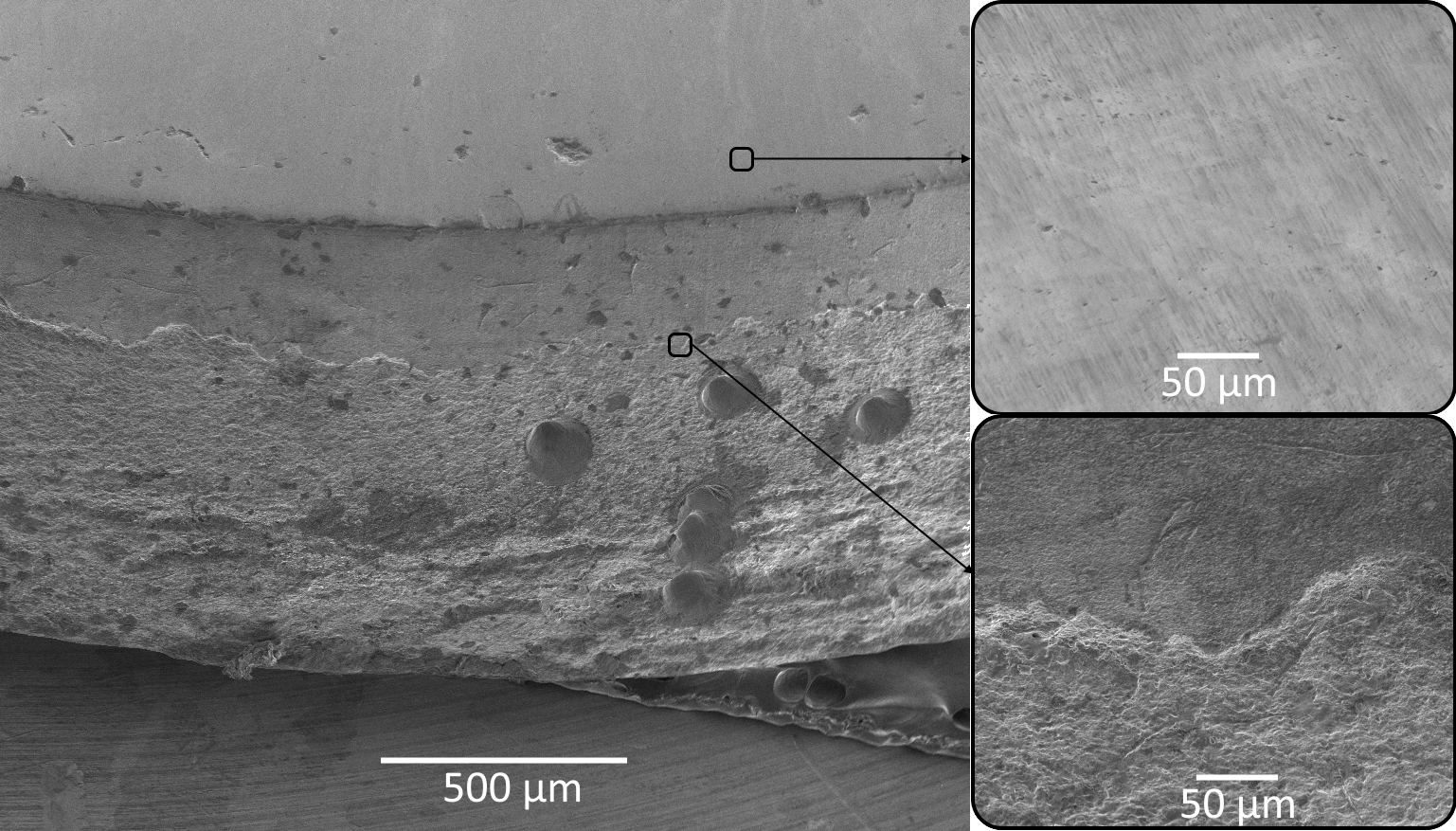
Fig. S6: SEM micrograph of the polished and unpolished surface of the transparent Cr:YAG ceramic.

Video. Cr:YAG ceramics under irradiation by 1064 nm focused laser beam in vacuum with the camera moving parallel to the side surface.
